# Peritoneal strumosis and the role of imaging in the diagnostic workup: A case report

**DOI:** 10.1016/j.radcr.2026.06.053

**Published:** 2026-07-04

**Authors:** Jiyeon Lee, Jeanette Meraz, Dillon Sommer, Jasmine Zhao, Ryan O’Connell, Roozbeh Houshyar

**Affiliations:** a School of Medicine, University of California, Irvine, CA, USA; b Department of Radiological Sciences, University of California Irvine, Orange, CA, USA; c Department of Pathology, University of California Irvine, Orange, CA, USA

**Keywords:** Peritoneal strumosis, Struma ovarii, Ovary

## Abstract

Peritoneal strumosis, also termed highly differentiated follicular carcinoma of ovarian origin (HDFCO), is a rare entity arising from struma ovarii, with extra-ovarian dissemination of thyroid tissue. Radiologic descriptions remain limited. We report a case of a 38-year-old woman with a known diagnosis of struma ovarii, a monodermal teratoma mainly of benign thyroid tissue, who presented with multiple peritoneal lesions detected on imaging. Magnetic resonance imaging (MRI) revealed diffusion-restricting enhancing soft tissue implants throughout the abdomen and pelvis, while nuclear medicine imaging demonstrated no I-123 radiotracer uptake. Biopsy confirmed well-differentiated thyroid tissue. This case highlights the role of MRI and nuclear medicine in identifying peritoneal strumosis and emphasizes the need to include this rare entity in the differential when radiologists encounter unexplained peritoneal nodules. Radiologists should consider HDFCO in the differential when encountering peritoneal nodules in patients with prior struma ovarii.

## Introduction

Peritoneal strumosis, recently reclassified by the World Health Organization as highly differentiated follicular carcinoma of ovarian origin (HDFCO), represents dissemination of thyroid tissue arising from struma ovarii (SO), an ovarian tumor with thyroid tissue arising from teratomas [1,2]. Although histologically benign, HDFCO demonstrates malignant behavior through its ability to spread beyond the ovary [2]. SO itself is rare, comprising < 5% of ovarian teratomas and < 1% of all ovarian tumors [1].

Imaging plays a central role in detection and surveillance of this disease. CT and ultrasound may show peritoneal or adnexal nodules, but findings can be nonspecific [1,3]. MRI provides superior soft tissue characterization, and radioiodine scans may be useful after thyroidectomy [4,5]. Peritoneal strumosis is exceptionally uncommon – ∼ 1.3% among patients with SO – with only 19 pooled cases in a 2023 systematic review; in that cohort, recurrence occurred in 2/19 patients, and no deaths were reported at a mean 53-month follow-up [1]. Ultimately, histopathologic confirmation is required. Here, we present the MRI and nuclear medicine findings of peritoneal strumosis with pathologic correlation.

## Case report

A 38-year-old woman initially presented with bloating and abdominal pain and was found to have large bilateral ovarian masses on outside imaging. She underwent resections at an outside institution resulting in mature teratomas. Her symptoms returned 3 years later with recurrent right ovarian lesion at an outside institution. Right salpingo-oophorectomy resulted in recurrent teratoma. Follow-up imaging at the outside institution showed multiple peritoneal masses that were biopsied and found to be strumosis without atypia. The timeline of important clinical events is outlined in Fig. 1.

**Fig. 1:**
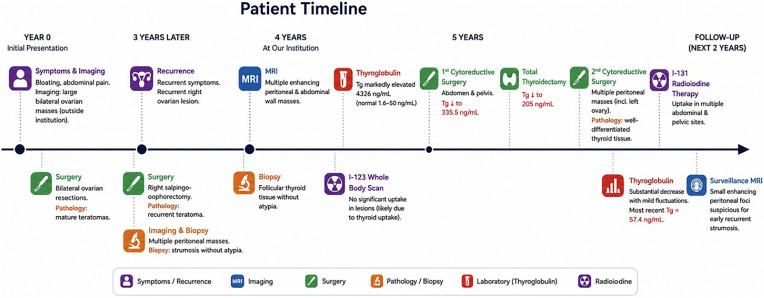
Timeline infographic of important clinical events.

Four years after her initial symptoms, she presented to our institution for additional workup and treatment due to relocation. MRI of the abdomen and pelvis demonstrated multiple enhancing peritoneal and abdominal wall masses and a homogenously enhancing structure in the left adnexa which was favored to represent the ovary (Fig. 2). The abdominal wall masses were biopsied and demonstrated follicular thyroid tissue without atypia. At this time, the patient had a markedly elevated thyroglobulin level of 4326 ng/mL (normal range 1.6-50 ng/mL). An I-123 whole body scan utilizing 1.2mCi of I-123 was administered to the patient, who was placed on a low iodine diet prior to the exam. The 24-hour uptake scan showed significant uptake in the thyroid, but no significant uptake in the multiple peritoneal and abdominal wall lesions. This finding may have been due to the uptake of the majority of the radiotracer by the thyroid gland (Fig. 4A).

**Fig. 2:**
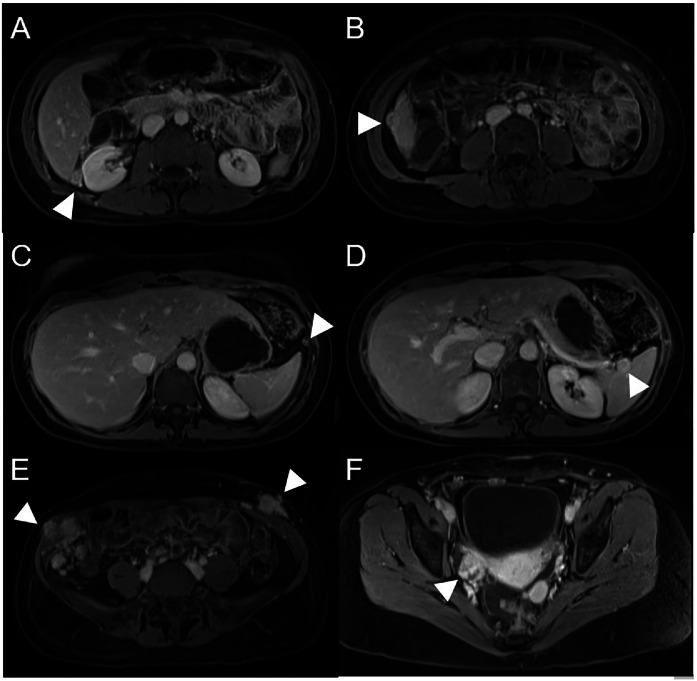
MRI of the abdomen and pelvis. Axial T1 weighted fat-suppressed post-contrast (gadoteridol 13 ml - 279.3 mg/mL) MR images demonstrate enhancing soft tissue lesions (white arrowheads) throughout the abdomen and pelvis, including the (A) hepatorenal space, (B) inferior right hepatic lobe, (C) left upper quadrant peritoneum, (D) splenic hilum, and (E) right paracolic gutter and bilateral ventral abdominal wall. Postsurgical changes of right salpingo-oophorectomy with enhancing structures along the (F) right pelvic sidewall/adnexa, which were favored to be another peritoneal deposit. The homogenously enhancing structure in the left adnexa was favored to represent the ovary.

Five years after her initial presentation, the patient underwent a round of cytoreductive surgery in the abdomen and pelvis, resulting in a lowering of the thyroglobulin level of 335.5 ng/mL. Several months after the cytoreductive surgery, she underwent total thyroidectomy, which further lowered the thyroglobulin level to 205 ng/mL. She then underwent a second round of cytoreductive surgery in the abdomen and pelvis with removal of multiple peritoneal masses including a mass in the left ovary. Pathological examination demonstrated well-differentiated follicular thyroid tissue (Fig. 3). The patient was reimaged using 3.4 mCi of I-123 (Fig. 4B), and a 24-hour uptake scan showed uptake in the remnant thyroid tissue in the thyroid bed. Shortly thereafter, given patient’s persistent elevated thyroglobulin levels, patient underwent radioiodine therapy with 128 mCi of I-131 with 7-day post ablation imaging demonstrating I-131 uptake within multiple sites in the abdomen and pelvis (Fig. 4C).

**Fig. 3:**
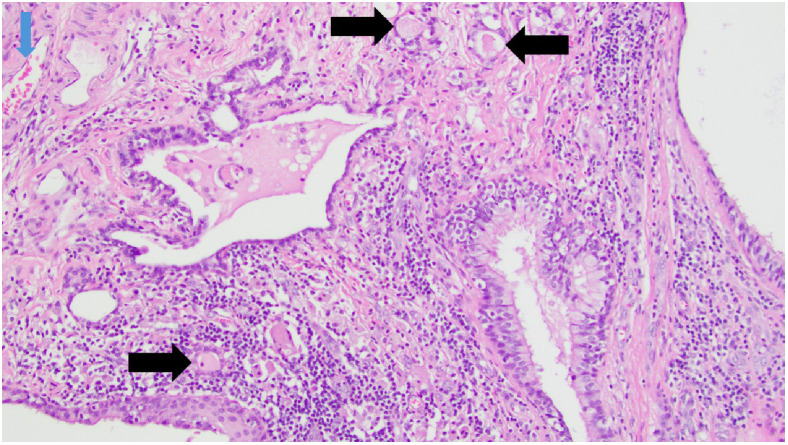
Pathology findings. This H&E-stained section (imaged at approximately 100x magnification) of a pelvic mass abutting the left ovary shows portions of dermoid cyst with scattered well-differentiated thyroid follicles. Occasional eosinophilic proteinaceous material is present within the follicles (black arrows). Intravascular red blood cells for scale (blue arrow).

**Fig. 4:**
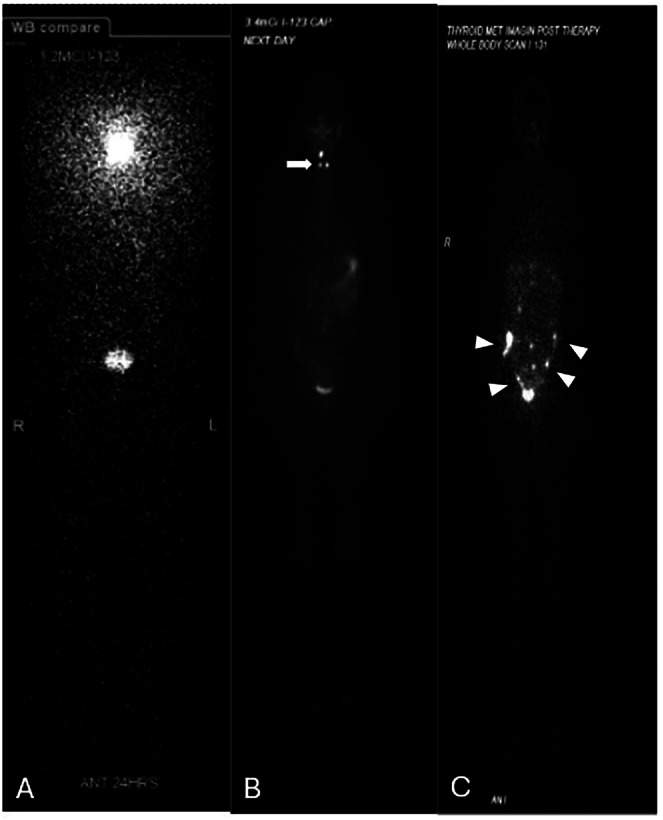
Initial Nuclear medicine I-123 uptake scan prior to thyroidectomy (A), post thyroidectomy 24-hour I-123 uptake scan post thyroidectomy (B) and post thyroidectomy I-131 ablation uptake scan (C). (A) Pretreatment scan shows significant uptake in the cervical thyroid but not in the abdominal and pelvic soft tissue lesions, likely due to majority of the radiotracer being taken up by the thyroid gland. Additional focus of uptake in the midline pelvis represents the bladder. (B) 24-hour post-thyroidectomy scan shows mild residual activity in the thyroid remnant (white arrow) without uptake in peritoneal lesions. (C) Follow up-post-thyroidectomy I-131 scan shows multiple foci of increased radiotracer activity seen in the abdomen and pelvis (white arrowheads) consistent with residual peritoneal metastases.

Over the next two years, her thyroglobulin levels decreased substantially with mild interval fluctuations, with the most recent level at 57.4 ng/mL. However, the most recent follow-up surveillance MRI showed small enhancing foci suspicious for early recurrent disease within the peritoneal cavity. There has been no post-surgical MRI or CT demonstrating a confirmed disease-free state during surveillance follow-up.

## Discussion

Peritoneal strumosis – also termed highly differentiated follicular carcinoma of ovarian origin (HDFCO) – is a rare entity characterized by extra-ovarian dissemination of thyroid tissue arising from struma ovarii [1,2]. Peritoneal strumosis itself is exceptionally uncommon – ∼ 1.3% among patients with struma ovarii, with only 19 pooled cases synthesized in a recent review – and recurred in a small minority (≈ 2/19) with long recurrence-free intervals [1]. Due to its rarity, the pathogenesis, standard treatment plan, or long-term prognosis of peritoneal strumosis cannot be determined, requiring highly individualized treatment plans. Of the known and published cases of peritoneal strumosis, surgery and radioiodine therapy are the initial treatment routes that are pursued with varying results [[1], [2], [3]]. While complete surgical excision has been documented to achieve disease control, recurrence rates remain substantial in malignant struma ovarii (21-35%), particularly in the absence of radioiodine therapy (RAI) [2,6]. Despite surgery and RAI, our patient demonstrated multiple recurrences, consistent with occasional reports of relapse even after definitive gynecologic surgery [2]. In this case, thyroidectomy was undertaken to eliminate competing physiologic thyroid iodine uptake, improve sensitivity of subsequent radioiodine imaging and therapy, and facilitate serial thyroglobulin surveillance.

From an imaging standpoint, peritoneal strumosis lesions are nonspecific and can mimic peritoneal carcinomatosis or recurrent ovarian carcinoma, which may present similarly as nodular enhancing thickening of the peritoneal serous layer, presence of ascites, and involvement of the greater omentum [7,8]. Features favoring peritoneal strumosis over peritoneal carcinomatosis include a more indolent clinical course, absence of aggressive ascites or diffuse omental caking, elevated thyroglobulin levels, and eventual iodine avidity on delayed nuclear medicine imaging [9,10]. Additional differential considerations include endometriosis and granulomatous peritoneal disease; however, the patient’s markedly elevated thyroglobulin levels, known history of struma ovarii, and eventual delayed radioiodine uptake favored HDFCO/peritoneal strumosis. The combination of diffusion-restricting nodules on MRI with absent uptake on I-123 scans should not exclude consideration of the diagnosis prior to biopsy confirmation. Paired cross-sectional imaging and targeted radioiodine studies help define the burden of disease and assess functional iodine-avid implants, which may be seen in HDFCO. For radiologists, recognizing this pattern – particularly the combination of peritoneal nodularity in a patient with prior struma ovarii – should prompt consideration of peritoneal strumosis, even in the absence of initial radioiodine uptake. As seen in our patient’s initial I-123 radioiodine uptake scan not showing uptake in the peritoneal masses, despite confirmed biopsy reports showing HDFCO, the absence of radioiodine uptake cannot definitively rule out the possibility of peritoneal strumosis as the diagnosis. In this case, the patient’s peritoneal masses showed radioiodine uptake with both I-123 and I-131 later in the disease progression. Based on our literature review, prior case reports on peritoneal strumosis have not reported an initial lack of radioiodine uptake. The initial absence of radiotracer uptake may reflect competitive physiologic thyroid uptake prior to thyroidectomy or variable iodine avidity of peritoneal implants, although the precise mechanism remains unclear.

Given the potential for indolent yet recurrent disease, serial multimodality surveillance (MRI/CT with selective nuclear medicine correlation) is warranted to document stability, detect new nodules, and guide timing of intervention [1,2,6]. Our patient demonstrated initial pathology consistent with peritoneal strumosis – thyroid follicular tissue with no atypia –, and over the following years, had multiple recurrences of both peritoneal strumosis and follicular thyroid carcinoma despite undergoing treatment. This suggests that, at a minimum, annual surveillance with imaging may be needed to assess new lesions. Additionally, routine follow-up is warranted to assess any new symptoms and follow thyroglobulin trends. Trending thyroglobulin and anti-thyroglobulin antibody has been used in some cases, and, in a prior case, has helped prove appropriate response to RAI treatment with no residual disease evidence [3]. However, in a study evaluating 18 patients, only 2 patients presented with elevated thyroglobulin levels, suggesting that trending thyroglobulin levels may only be of use in cases with initial elevated thyroglobulin [1].

## Conclusion

Peritoneal strumosis, or HDFCO, is a rare and incompletely understood entity with potential for recurrence even after multimodal treatment. In this case, serial MRI, CT, and nuclear medicine studies were essential for documenting disease progression and guiding management over several years. Given the absence of standardized treatment protocols, individualized therapy is required, but consistent multimodality imaging follow-up remains critical for early detection of recurrence, accurate differentiation from malignancy, and surgical planning.

## Patient consent

Written informed consent was obtained from the patient for publication of this case report, including any accompanying images.

## References

[1] Li S., Wang X., Hong R., Zhang X., Yin M., Zhang T. (2023). Clinical characteristics, treatment, and outcomes of peritoneal strumosis: A report of three cases and systematic review. Diagnostics (Basel).

[2] Giovannopoulou E., Saliaris K., Kavoura E., Pavlakis K., Lathouras K. (2022). Highly differentiated follicular carcinoma of ovarian origin: a systematic review of the literature. Curr Oncol.

[3] Yang J., Pan Z., Wang J., Su X. (2025). Complete remission of peritoneal strumosis from struma ovarii after radioiodine therapy: a case report. Thyroid Res.

[4] Groener D., Baumgarten J., Happel C., Mader N., Ngoc C.N., Sabet A. (2023). Thyroid papillary cancer elements arising from struma ovarii with benign peritoneal strumosis: utility of iodine-123 imaging in diagnostics and treatment planning. Clin Case Rep..

[5] Asaturova A., Magnaeva A., Tregubova A. (2022). Malignant clinical course of ``proliferative'' ovarian struma: diagnostic challenges and treatment pitfalls. Diagnostics (Basel).

[6] Kantreva K., Paschou S.A., Stefanaki K., Pappa K., Kazakou P., Vrachnis D. (2024). Two rare cases of benign struma ovarii with malignant recurrence. Endocrinol Diabetes Metab Case Rep.

[7] Diop A.D., Fontarensky M., Montoriol P-F, Da Ines D. (2014). CT imaging of peritoneal carcinomatosis and its mimics. Diagn Interv Imaging.

[8] Miguez González J., Calaf Forn F., Pelegrí Martínez L., Lozano Arranz P., Oliveira Caiafa R., Catala Forteza J. (2023). Primary and secondary tumors of the peritoneum: key imaging features and differential diagnosis with surgical and pathological correlation. Insights Imaging.

[9] Levy A.D., Shaw J.C., Sobin L H (2009). Secondary tumors and tumorlike lesions of the peritoneal cavity: imaging features with pathologic correlation. Radiographics.

[10] Roth L.M., Karseladze A I (2008). Highly differentiated follicular carcinoma arising from struma ovarii: a report of 3 cases, a review of the literature, and a reassessment of so-called peritoneal strumosis. Int J Gynecol Patholo.

